# Modulation of motor excitability by cortical optogenetic theta burst stimulation

**DOI:** 10.1371/journal.pone.0203333

**Published:** 2018-08-30

**Authors:** Chun-Wei Wu, Wen-Tai Chiu, Tsung-Hsun Hsieh, Cho-Han Hsieh, Jia-Jin Jason Chen

**Affiliations:** 1 Department of Biomedical Engineering, National Cheng Kung University, Tainan, Taiwan; 2 School of Physical Therapy and Graduate Institute of Rehabilitation Science, Chang Gung University, Taoyuan, Taiwan; 3 Neuroscience Research Center, Chang Gung Memorial Hospital, Linkou, Taoyuan, Taiwan; Shanghai Mental Health Center, CHINA

## Abstract

Intermittent theta burst stimulation (iTBS) and continuous theta burst stimulation (cTBS) are protocols used in repetitive transcranial magnetic stimulation (rTMS) or cortical electrical stimulation (CES) to facilitate or suppress corticospinal excitability. However, rTMS and CES excite all types of neuron in the target cortex probed by the coil or electrode, making it difficult to differentiate the effect of TBS on specific neural circuits involved in motor plasticity. In this study, TBS protocols were converted into an optogenetic model to achieve focalized and cell-type-specific cortical modulation. Light-sensitive channelrhodopsin-2 (ChR2) was expressed in the glutamatergic neuron in the primary motor cortex (M1) driven by the CaMKIIα promoter. A custom-made optrode comprising an optical fiber and a metal cannula electrode was fabricated to achieve optogenetic stimulation and simultaneous local field potential (LFP) recording. Single-pulse CES was delivered into M1 to elicit motor-evoked potential (MEP), which served as an indicator of motor excitability, before and after TBS intervention. Results show that both CES-iTBS and optogenetic iTBS (Opto-iTBS) can potentiate MEP activity. However, CES-cTBS suppressed MEP activity whereas Opto-cTBS enhanced it. This discrepancy may have resulted from the different neural networks targeted by the two TBS modalities, with CES-cTBS exciting all types of neuron and Opto-cTBS targeting excitatory neuron specifically. The results support the idea that intra-cortical networks determine the variation of TBS-induced neuroplasticity. This study shows that focalized and cell-type-specific brain stimulation using the optogenetic approach is viable and can be extended for further exploration of neuroplasticity.

## Introduction

By generating electrical current inside the brain, repetitive transcranial magnetic stimulation (rTMS) and transcranial electrical stimulation can modify brain plasticity, in a manner similar to the *in vitro* induction of long-term potentiation (LTP) and depression (LTD) [1,2]. Depending on various stimulation parameters (frequency, duration, and amplitude) and patterns, such non-invasive brain stimulation (NIBS) is capable of producing long-lasting facilitation or suppression of cortical excitability, with various behavioral and psychological effects. Among various stimulation forms of NIBS, theta burst stimulation (TBS) produces a very powerful and reproducible effect and has thus attracted increasing attention [3–6]. By mimicking the coupling of theta and gamma rhythms in rodent electroencephalograms during learning and exploration, electrical TBS was originally applied to the hippocampus and motor cortex to induce LTP in brain slice studies [7,8]. Using rTMS, TBS protocols can induce LTP- and LTD-like effects in the human motor cortex [4,6]. The modulating effects of TBS on motor excitability was determined according to the changes in single-pulse TMS-induced electromyographic (EMG) responses, also known as motor-evoked potentials (MEPs). Huang et al. found that MEPs were enhanced after intermittent TBS (iTBS) but suppressed after continuous TBS (cTBS). Related studies confirmed the effectiveness of iTBS and cTBS in modulating motor plasticity in healthy human subjects and rats [9,10]. ITBS and cTBS patterns determine the direction of change in motor excitability and thus require shorter stimulation duration (192 s for iTBS; 40 s for cTBS) and lower intensity to induce longer-lasting effects compared to those for conventional repetitive stimulation protocols such as low-frequency (≤ 1 Hz) and high-frequency (≥ 5 Hz) stimulation. These properties make iTBS and cTBS more acceptable for human subjects and accessible for non-anesthetized animals.

TBS produces a strong effect and bidirectional plasticity changes, making it a potential option for diagnosis and therapy for many neurological movement disorders, such as amyotrophic lateral sclerosis, multiple sclerosis, and stroke [3]. However, the mechanism of the modulation of brain plasticity induced by TBS is still not fully understood. Epidural recordings at a high cervical level suggest that rTMS-iTBS and -cTBS regulate different interneuron networks connected to corticospinal neurons [5,11]. In the case of modulation of synaptic plasticity, the parameters of the stimulation, such as temporal precision, special localization, and cellular specificity, in a neural network are important [12]. According to studies [5,11] using epidural recordings, it is essential to investigate the *in vivo* mechanisms of iTBS and cTBS by manipulating neural activity in a cell-type-specific way. A previous rodent study demonstrated that rTMS-TBS protocols are applicable for studying impaired motor plasticity in hemiparkinsonian rats [10]. Due to the relatively large size of the coil compared to that of the rat brain, the stimulation was not limited to the motor cortex. Cortical electrical stimulation (CES) delivered via microelectrodes has better spatial precision [13], but it cannot target specific a synapse, subcellular location, or cell type. Both TMS and CES are quite non-focal and affect neural pathway interactions, which makes their results unpredictable.

The above limitations may be overcome using an emerging technique called optogenetics, which utilizes *in vivo* tissue-specific expression of light-sensitive channels (opsins) and allows specific neuron populations to be selectively activated or inhibited by certain wavelengths of light with millisecond temporal precision [14–16]. Because of its specificity and spatiotemporal precision, optogenetics is widely used to study the neural substrates underlying complex animal behavior [17]. In the present study, we investigate the role of excitatory neuron in motor plastic modulation by TBS protocols using optogenetic stimulation. CaMKIIα-promoter-driven channelrhodopsin-2 (ChR2) was expressed in glutamatergic pyramidal neurons in the primary motor cortex (M1) of rats. ChR2-mediated neural excitation was induced with blue laser light (473 nm) irradiation [15,18]. We designed a novel implantable optrode to guide the blue laser light into brain tissue and simultaneously collect local field potentials (LFPs) evoked by optogenetic stimulation to confirm the success of ChR2 expression. CES-induced MEPs were measured before and after TBS treatment to determine the modulating effect of TBS on motor excitability.

## Materials and methods

### Animal preparation

All animal experiments were approved by the National Cheng Kung University Medical College Animal Use Committee (IACUC Approval No.: 104139). Adult male Sprague Dawley rats, weighing 300–350 g, were housed in standard cages at a temperature of 25 ± 1°C with a 12/12-h light/dark cycle and free food and water access.

### Lentivirus production

The lentiviral vector carrying channelrhodopsin-2, pLenti-CaMKIIa-hChR2(H134R)-EYFP-WPRE [19], was gifted by Karl Deisseroth from the Optogenetics Resource Center, Stanford University, Stanford, CA, USA (Addgene plasmid #20944). The construct was packaged in a second-generation lentivirus system via calcium-phosphate co-transfection of 293FT cells with psPAX2 and pMD2.G (gifted by Dr. Didier Trono, Addgene plasmids #12260 and #12259). The viral pellet was concentrated and then resuspended in phosphate-buffered saline (PBS) at 1/1000^th^ of the original volume. To determine the infection titer of the viral solution, a neuroblastoma cell line, SH-SY5Y, was infected by the lentivirus at serial dilution concentrations. Seventy-two hours after transduction, the number of eYFP positive cells was counted using fluorescence microscopy. The infection unit (IU) of the viral solution was determined as: # of eYFP-positive cells/ml of viral solution.

### Optrode design

**Fig 1:**
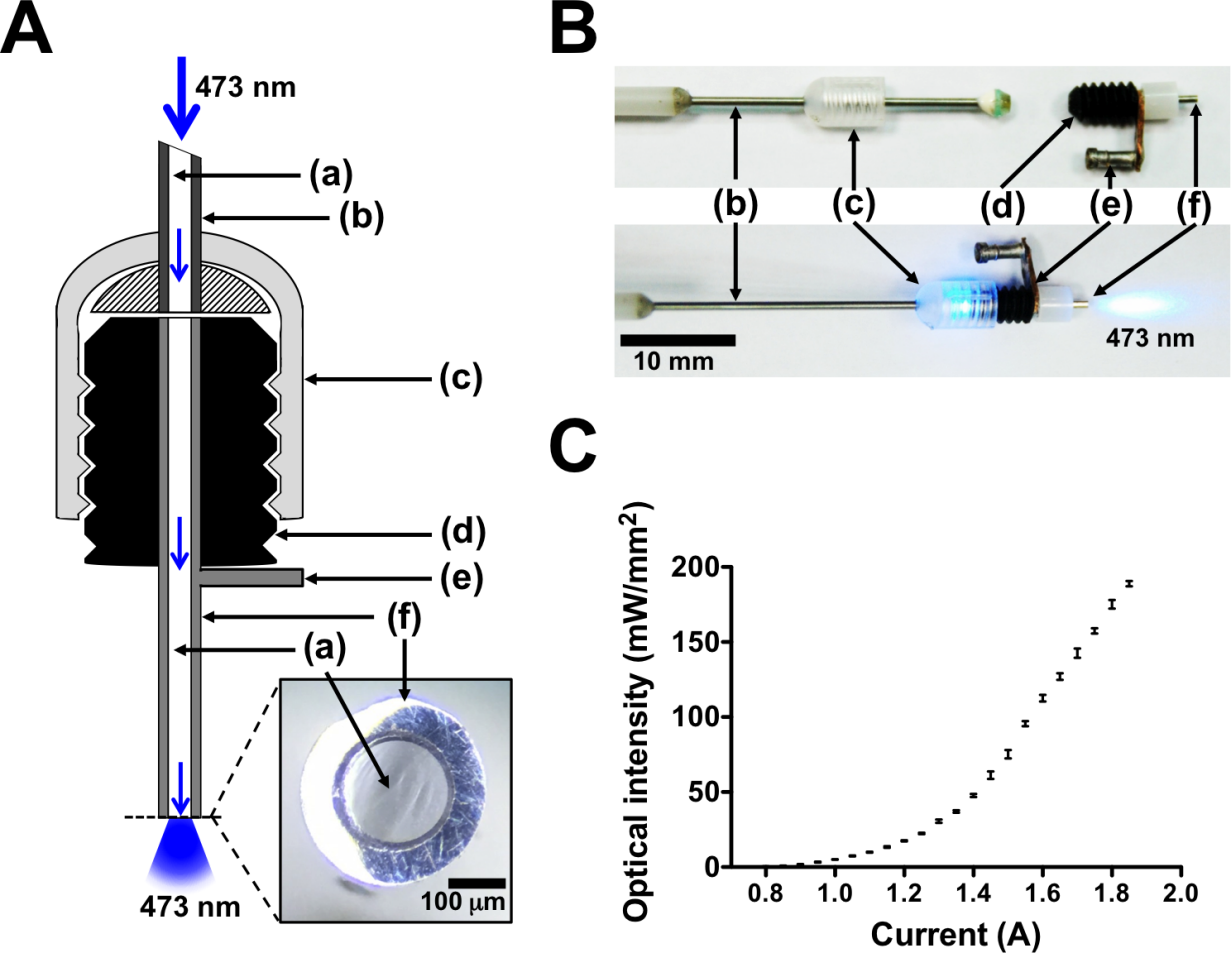
Optrode design for simultaneous optogenetic stimulation and LFP recording. **A** Schematic diagram of fiber-based opto-electrical neural interface showing (a) optical fiber, (b) fiber housing, (c) fiber cap, (d) pedestal of optrode, (e) connector for CES or LFP recording, and (f) stainless steel cannula (serving as cortical electrode). Close-up schematic (bottom right) shows polished surface of optrode tip. **B** Connection of optrode to patch cord fiber. **C** Optical intensity curve. Power density of blue light emitted from optrode is plotted (mean ± SD) versus laser-driven current.

An integrated optrode was designed to achieve *in vivo* chronic neural recoding, CES, and optical stimulation (Fig 1A). An optical fiber (BFH22-200, Thorlabs) with a 200-μm-diameter core was inserted into a stainless steel cannula pedestal (C200GS-5/SPC, Plastics One) and glued to it. Both ends of the fiber were polished to avoid light scattering due to a rugged surface. The stainless steel cannula was then wired to a connector to serve as a CES and neural recording electrode. A fiber optic patch cord (core diameter: 200 μm) was fabricated with an FC connector (30230G3, Thorlabs, Inc.) on one end, and a custom-made connector on the other end, which consisted of a fiber housing (18G syringe needle) and a fiber cap (303/OFC, Plastics One). During optical stimulation, the patch cord bridged the 473-nm laser (MBL-III-473-50, Ultralasers Inc.) and the optrode (Fig 1B). The intensity of the blue laser light emitted from the tip of the optrode was measured (NOVA-II, Ophir Optronics) and plotted versus driving current (Fig 1C).

### Virus injection and optrode implantation

**Fig 2:**
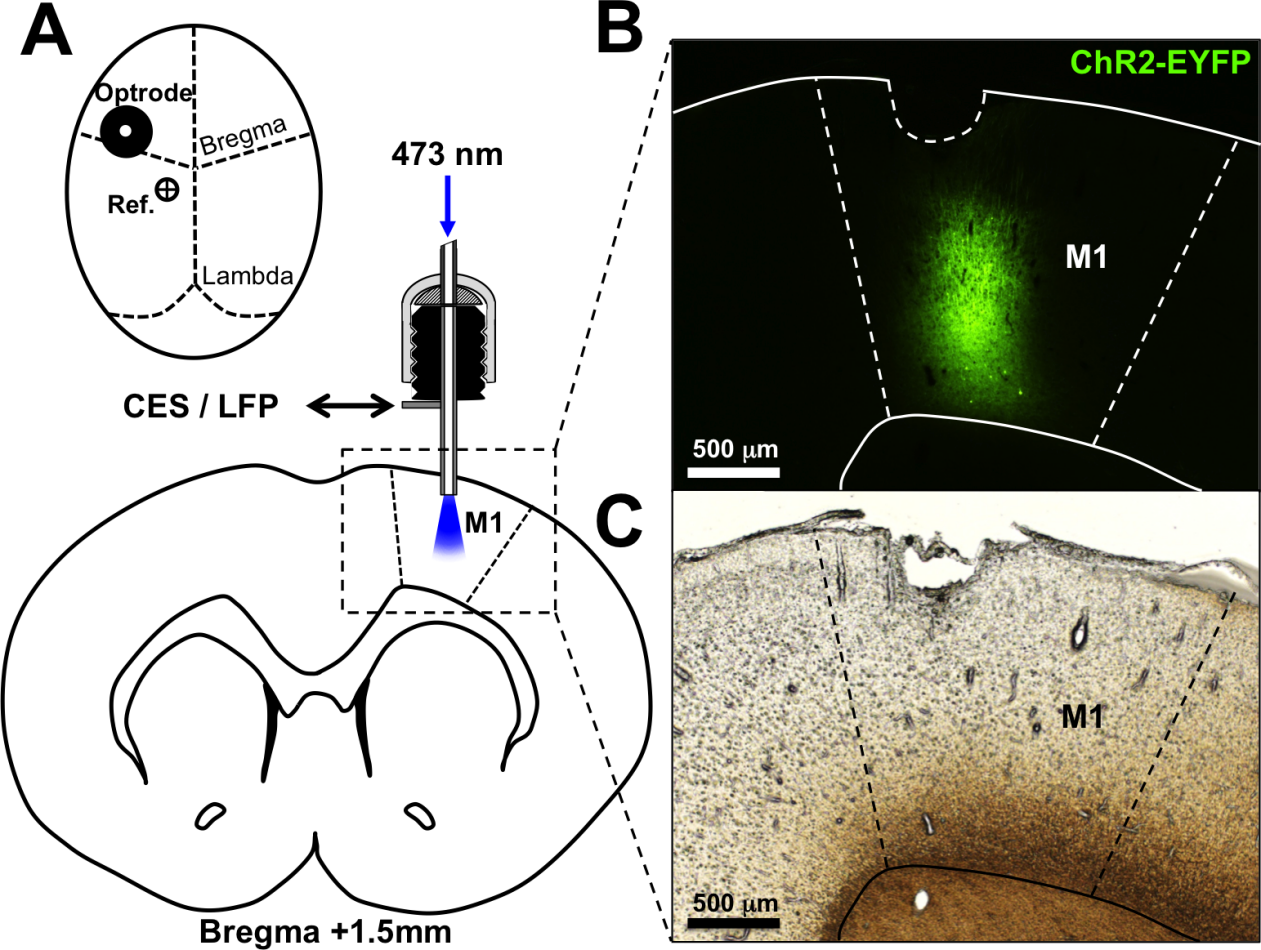
Implantation of optical neural interface into rat’s brain. **A** Schematic of optrode and reference electrode mounted on rat skull corresponding to M1 forelimb area. Lentivirus carrying ChR2 gene fused to eYFP under control of CaMKIIα promoter was injected into M1 right before optrode implantation. **B** Close-up images of intrinsic eYFP fluorescence and **C** bright-field image of acute brain slice showing rat M1. ChR2-eYFP was expressed at layer 2 through layer 6. Insertion of optrode caused lesion on cortex surface, which indicates position of implantation.

Rats were introduced anesthetization with inhalation of 4% isoflurane in O_2_, then placed in a stereotaxic apparatus (Model 902, DAVID KOPF INSTRUMENTS) and maintained anesthetization using 2.5% isoflurane inhalation. The scalp was shaved and sterilized, then an incision was made on the scalp to expose the skull and bregma after the soft tissue had been carefully removed. A burr hole was drilled and 2 μl of concentrated lentivirus solution (10^7^ IU/ml) was injected into the left primary motor cortex of the right forelimb (AP: +1.5 mm; ML: 2.5 mm; DV: 1.5 and 2.5 mm) at a rate of 0.1 μl/min controlled by a syringe pump (KDS 100, KD Scientific Inc.). After injection, the optrode was implanted at the same site for future optical and electrical stimulation and neural recording (Fig 2A). A reference stainless steel screw electrode (3.0 mm × 1.4 mm) was implanted into the left M1 hindlimb (AP: -1.0 mm and ML: 1.25 mm) according to functional brain mapping of rats [20]. Both the optrode and the reference screw were connected to a miniature socket, which was exposed for electrical stimulation and neural recording. After implantation, the electrodes and sockets were covered with dental cement. To relief pain after surgery, buprenorphine (0.1mg/kg) was injected subcutaneously every 12 hours for a week. Experiments began 4 weeks later to allow ChR2 expression.

### Optical stimulation and evoked potential recording

The rats were anesthetized using i.p. zoletil 50 (50 mg/kg, Virbac) mixed with xylazine (10 mg/kg, Bayer). Thirty minutes after anesthetization, the evoked potential induced by optogenetic stimulation was measured from ChR2+ neurons. A 473-nm diode-pumped laser (MBL-III-473-50, Ultralasers Inc.) was controlled by a pulse generator (Model 2100, A-M Systems). Blue light was guided into M1 optrode through a patch cord fiber. The LFP activity collected by the optrode was amplified (20,000×) and filtered using 60-Hz notch and 10 Hz to 600 Hz bandpass filters with a sampling rate of 10 kHz (MP36, BIOPAC Systems Inc.). During the recording, a marker synchronized with the pulse was sent simultaneously from the pulse generator to the data acquisition unit (MP36, BIOPAC Systems Inc.). The classic waveform of the evoked potential was obtained by applying coherent averaging to a hundred epochs according to the marker. The peak-to-peak amplitude was quantified for representing corticospinal excitability.

### Measurement of MEP

MEP was quantified following the protocol described by Hsieh et al. with several modifications [13]. The rats were anesthetized using i.p. zoletil 50 (50 mg/kg, Virbac) mixed with xylazine (10 mg/kg, Bayer). Thirty minutes after anesthetization, EMG activity from the belly of the right brachioradialis muscle was recorded with needle electrodes (Axon Systems, Inc.). A reference electrode was inserted into the right paw and a ground electrode was inserted into the base of the rat’s tail. MEP was elicited by applying an electrical pulse (biphasic, amplitude: 5 ~ 8V, pulse duration: 0.6 ms, pulse interval: 10 s; Model 2100, A-M Systems) over M1 via the stainless steel cannula of the optrode. The EMG signal was amplified 1000-fold and filtered using 60-Hz notch and 10 Hz to 1 kHz bandpass filters prior to digitization at a sampling rate of 10 kHz (MP36, BIOPAC Systems Inc.). The minimal intensity of a single biphasic pulse required to induce a MEP of greater than 20 mV was defined as the resting motor threshold (RMT). To quantify MEP, 30 trials at 120% RMT intensity were conducted to obtain the average of peak-to-peak amplitude for each MEP.

### Experimental design

Before the experiment of MEP modulation by TBS, all of the rats were tested to confirm the successful expression of the ChR2 by measuring the evoked potentials, and successful induction of MEP by determining the RMT. Only the rats with positive expression of ChR2 and MEP were included in the TBS experiment (n = 7 rats).

The basic pattern of TBS consisted of bursts that repeat at 5 Hz, and each burst is composed of three pulses that repeat at 50 Hz. Two types of TBS protocol were used in this study. For the cTBS protocol, the 5-Hz bursts were given in a continuous train and lasted for 40 s (total of 600 pulses). For the iTBS protocol, a 2-s train of TBS followed by 8 s of rest was repeated for 20 cycles (192 s, total of 600 pulses) [4,10,13]. The TBS protocols were generated by the pulse generator and converted into optical pulses (pulse width: 1 ms, intensity: 100 mW/mm^2^) via a transistor-transistor-logic-controlled diode-pumped laser (Opto-iTBS and Opto-cTBS). For sham control intervention, no stimulation was delivered. To study the effect of optogenetic TBS on motor excitability, the MEPs were measured at 10 minute before (baseline) and through 30 minutes after cortical TBS treatment. Every 5 minutes of MEPs (0.1 Hz, 30 trials) were collected for averaging. To compare the effects of TBS intervention on MEPs, all averaged MEP amplitudes were normalized to the baseline. Five types of TBS treatments have been performed on each rat: sham control, CES-iTBS, CES-cTBS, Opto-iTBS, and Opto-cTBS. Three to five days of resting between each experiment allowed elimination of any toxic effect of anesthetizer and accumulated effect of TBS-modulation.

### Histological examination

The intrinsic fluorescence of the ChR2-eYFP fusion protein was monitored to confirm the expression of ChR2. The rats were sacrificed via transcardial perfusion with ice-cold saline followed by 4% paraformaldehyde (PFA) in PBS. Extracted brains were further fixed in 4% PFA/PBS overnight, and then dehydrated in 30% sucrose/PBS for two days. Using a freezing microtome (Shadon Cryotome E, Thermo Electron Corp.), 30-μm sections were cut from optimal cutting temperature (OCT) compound-embedded brain tissue. Fluorescence images (excitation: 460–495 nm, emission: 510–550 nm) of ChR2-eYFP-positive neurons were observed under a microscope (IX-71, Olympus Corp.).

### Data analysis

All data are presented as means ± standard error of the mean (SEM). Data were analyzed using GraphPad Prism (version 5.01, GraphPad Software) with significance set to *p* < 0.05. Linear regression analysis was performed to test the dose effects of power density (mW/mm^2^, on logarithmic scale) on the amplitude of optogenetic-evoked potential. To compare the effects of various pulse widths of optogenetic stimulation on LFP, the peak-to-peak amplitudes of LFPs were normalized with respect to that amplitude at 1.0 ms. Kruskal-Wallis test with Dunn’s post hoc test were applied to compare the differences between various pulse width groups. Non-parametric multiple comparisons were performed to analyze the effects of two factors on MEP activities separately: 1) type of treatment (CES-iTBS/cTBS, Opto-iTBS/cTBS, and sham) and 2) time-course (10 and 5 min before and 5, 10, 15, 20, 25, and 30 min after TBS intervention). The MEPs were normalized to the baseline recorded at 10 min before treatment (-10 min). First, Kruskal-Wallis test was used to analyze the difference between iTBS versus sham and cTBS versus sham at each time-course. Second, Friedman’s test was used to exam the difference between each time-courses versus baseline (-10 min) under individual treatments (CES-iTBS/cTBS and Opto-iTBS/cTBS). The significant differences between groups were tested by post hoc Dunn’s test, and marked as: ^#^*p* < 0.05 versus sham; *p < 0.05 versus baseline (-10 min).

## Results

### Optogenetic-evoked potentials

A novel opto-electrical neural interface (Fig 1) was developed to activate M1 and simultaneously record the neural response. The optrode consisted of an optical fiber inserted in a stainless steel cannula, which was embedded in a mounting pedestal. The interface serves three purposes: (i) optical stimulation via the optical fiber and (ii) CES and (iii) cortical recording via the stainless steel cannula. The optrode was implanted right after lentiviral injection. With the identification of intrinsic fluorescence, expression of the CaMKIIα-promoter-driven ChR2-eYFP was confirmed in the forelimb area of M1 (Fig 2B and 2C).

**Fig 3:**
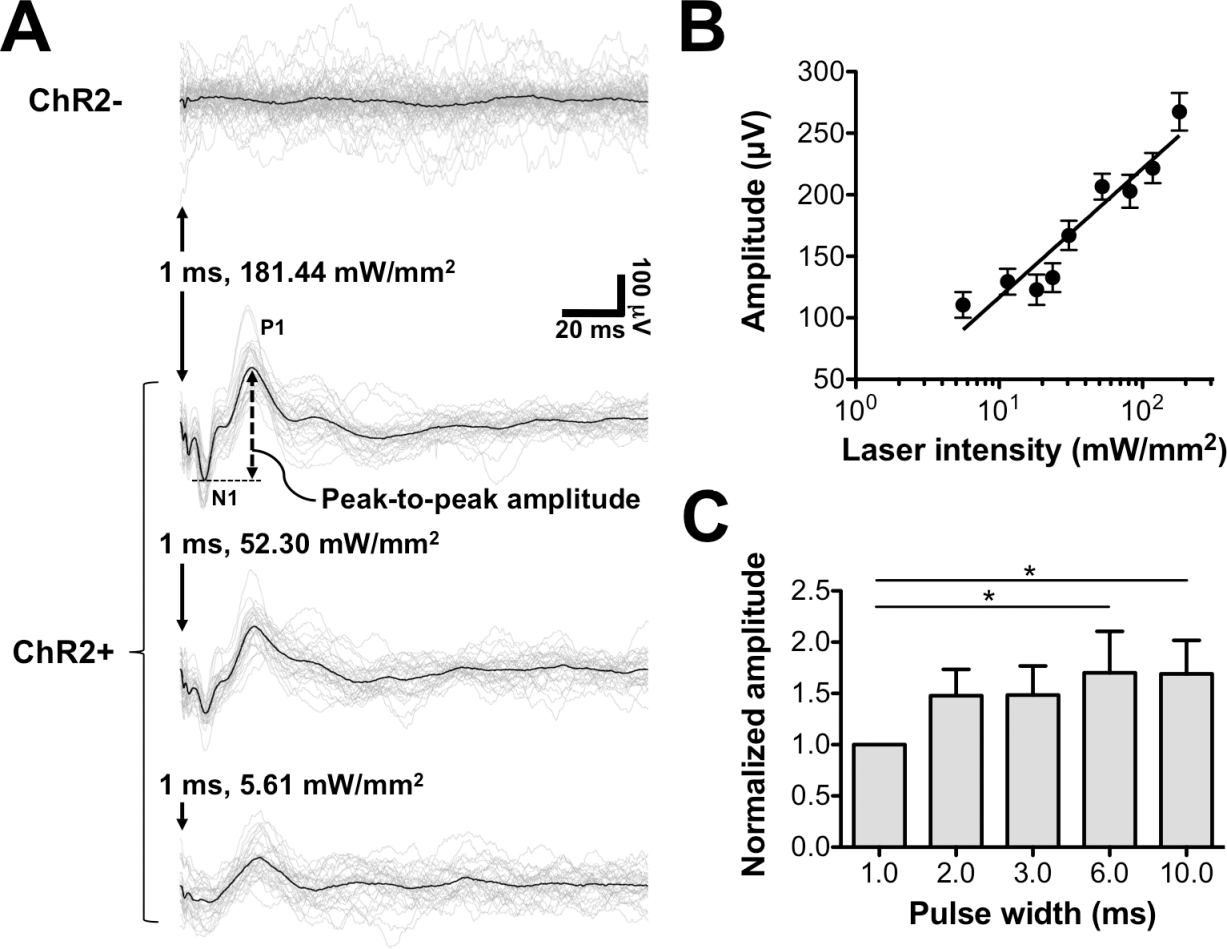
Recordings of optogenetic-evoked potentials under various laser intensities and pulse widths. **A** Representative LFP traces (grey line) and averaging waveform (black line) recorded during 1-ms single-pulse optical stimulation in ChR2-negative (ChR2-) and ChR2-positive (ChR2+) rats. Light-induced artifacts in LFP traces (marked with ↓) can be observed. Peak-to-peak amplitudes were measured as potential difference between N1 and P1. **B** Dose-response curve of peak-to-peak amplitudes evoked by various power densities on logarithmic scale (log10). Linear least squares fitting curve is plotted in solid line (R^2^ = 0.91). Each point corresponds to mean of amplitudes (μV) ± standard error of the mean. **C** Amplitudes evoked by 100-mW/mm^2^ optical pulses with various durations. Each bar corresponds to mean of normalized LFP amplitude ± standard error of the mean. *p < 0.05.

To activate ChR2, various optical intensities of the blue laser were emitted into the cortex. Representative LFP traces obtained for various intensities of single-pulse optical stimulation are shown in Fig 3A. Light-induced artifacts caused by the photoelectric effect appear in the traces for both ChR2-negative (ChR2-) and ChR2-positive (ChR2+) rats. A comparison to ChR2-negative traces allowed the typical waveform of optogenetic-evoked potentials induced by a 1-ms laser pulse to be identified in the traces from ChR2-positive M1. Representative waveforms induced by laser light at three optical intensities (5.61, 52.30, and 181.44 mW/mm^2^) were plotted after coherent averaging. The average latencies of N1 and P1 are 8.92 ± 0.66 ms and 24.38 ± 0.89 ms, respectively. The dose-response curve of the amplitudes elicited by various power densities are shown in Fig 3B. A linear dependency is observed (R^2^ = 0.91) between the peak-to-peak amplitudes of the evoked potential and optical intensity on the logarithmic scale. The effect of pulse duration on the amplitude of the evoked potential was also tested. A curve of evoked potential versus optical pulse duration is shown in Fig 3C. Significant differences between the evoked potential for 1.0 ms and those at other stimulator durations were observed in the Kruskal-Wallis test with Dunn’s post hoc test (*p* < 0.05).

### Effects of TBS on MEP activity

**Fig 4:**
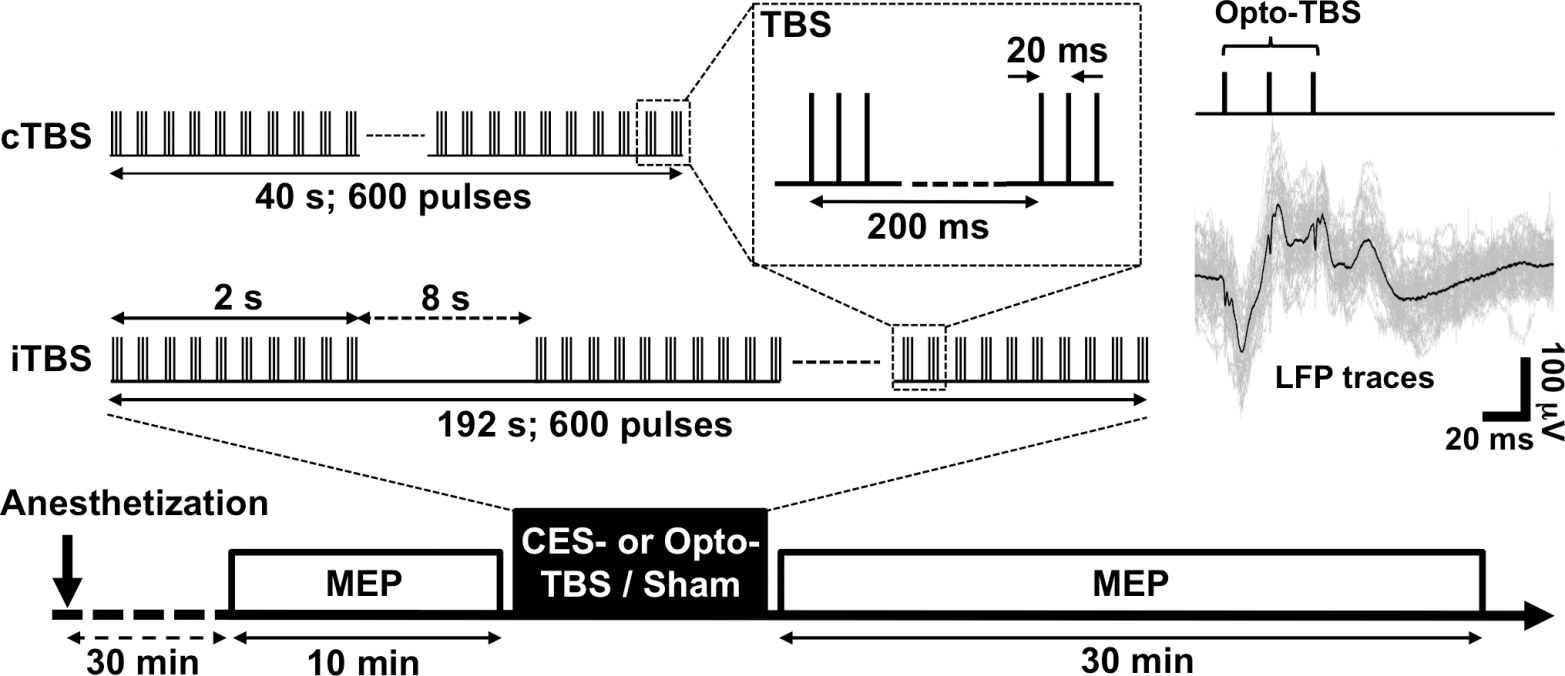
Experimental design of CES- and Opto-iTBS/cTBS protocols applied to ChR2-expressed rats. Averaged LFP trace is shown as black line in top-right schematic, demonstrating response of ChR2-positive M1 during Opto-TBS intervention. Thirty minutes after full anesthetization, MEPs were recorded for 10 min as baseline. After TBS intervention, 30-min MEPs were recorded for comparison with baseline. Total of five sets of experiments were performed, including CES-iTBS, CES-cTBS, Opto-iTBS, Opto-cTBS, and sham (no stimulation) sessions.

The MEP activities were recorded to evaluate the after-effects of sham, iTBS, and cTBS interventions applied in the forms of CES and optogenetic stimulation (Fig 4). The waveform of evoked potential corresponding to optogenetic theta burst stimulation (Opto-TBS) appears in the averaged LFP trace shown in Fig 4. This indicates the feasibility of using Opto-TBS intervention for cortical stimulation.

**Fig 5:**
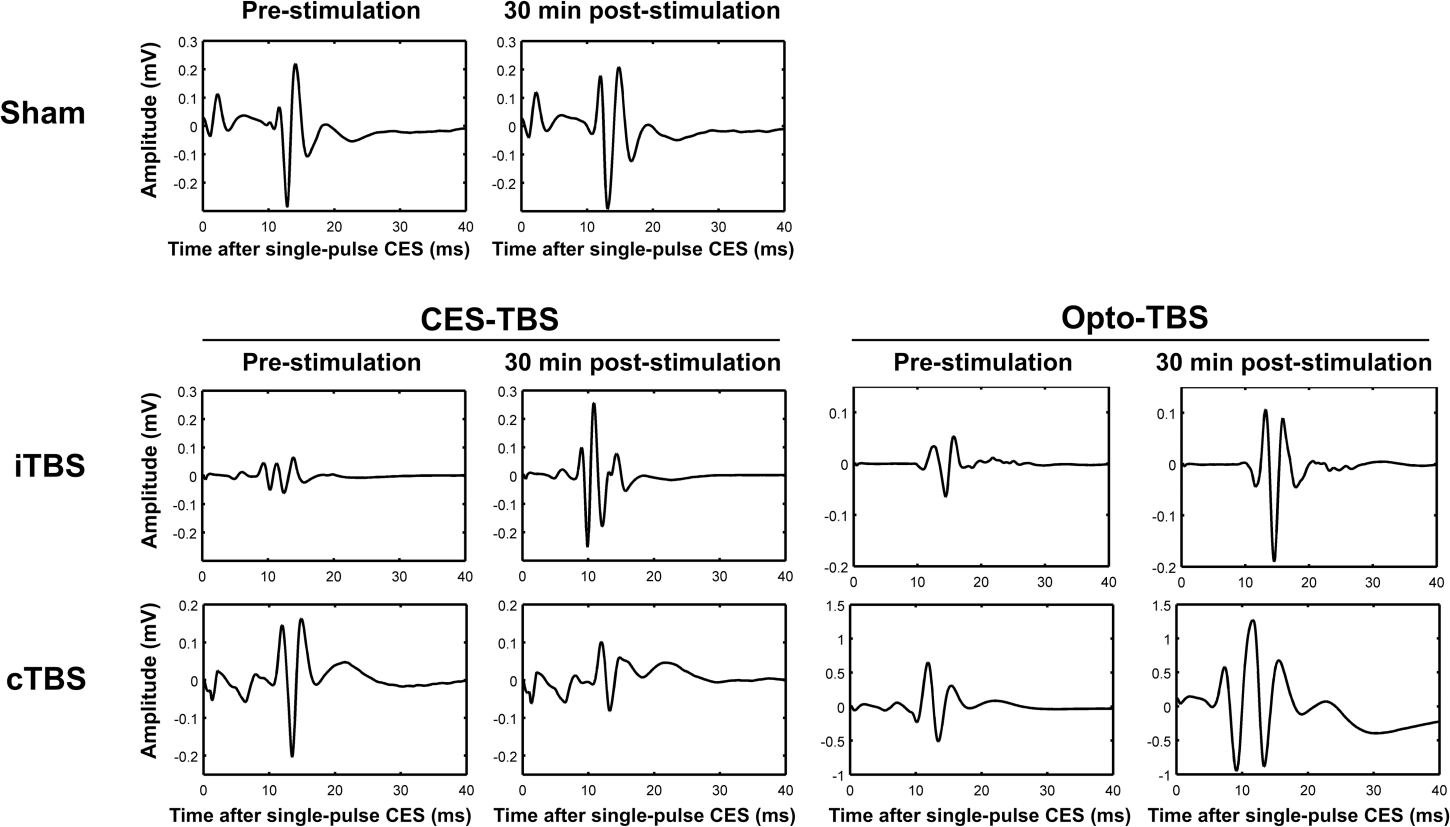
Representative MEP traces before and 30 min after sham, CES-iTBS, CES-cTBS, Opto-iTBS, and Opto-cTBS treatments. MEP waveforms appear within 10 to 20 ms of EMG signal after single-pulse CES. No obvious change occurred after sham stimulation. MEP traces show increased amplitude after CES-iTBS, Opto-iTBS, and Opto-cTBS treatments, and reduced amplitude after CES-cTBS.

**Fig 6:**
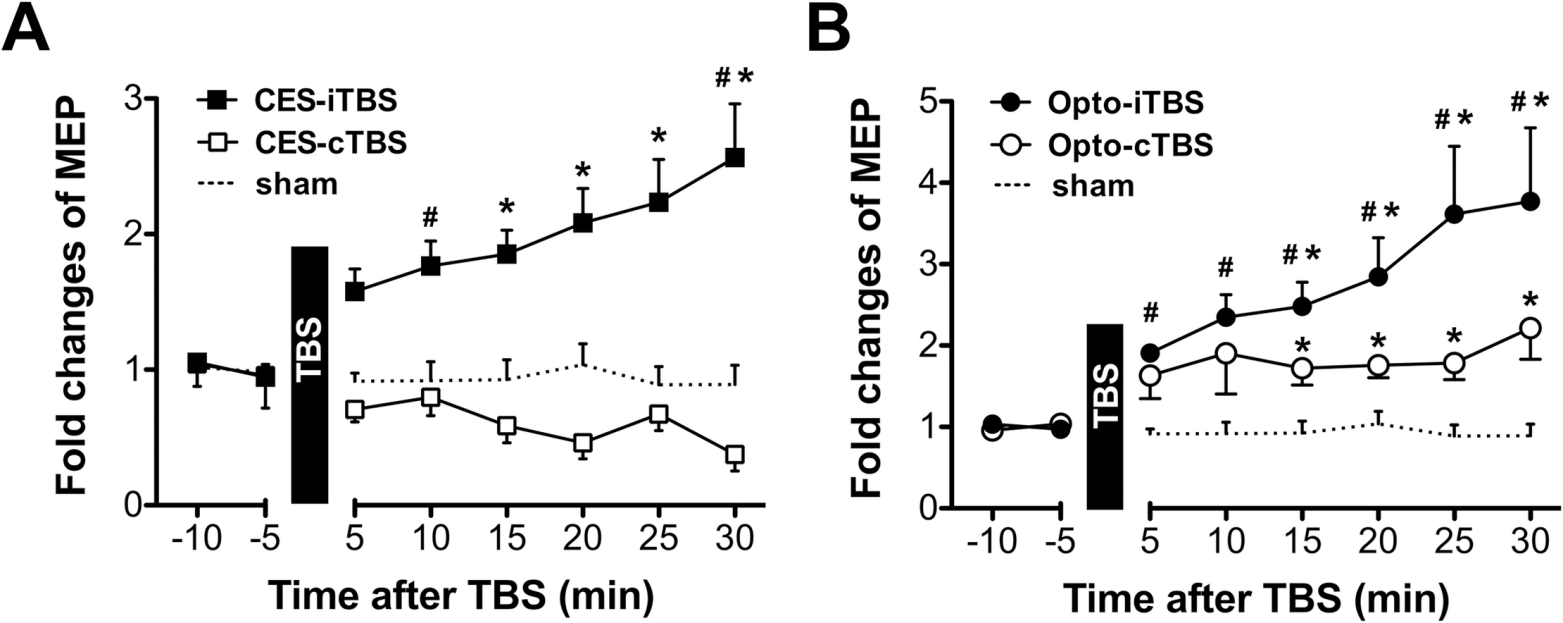
Effects of TBS on MEPs. Fold changes were observed in MEP amplitude after **A** CES-iTBS/-cTBS and **B** Opto-iTBS/-cTBS interventions. Each data point corresponds to average ± standard error of the mean in normalized MEP amplitude. The significant differences were marked as #*p* < 0.05 versus sham; *p < 0.05 versus baseline (-10 min) by post hoc Dunn’s test.

Representative EMG traces recorded 10 min before and 30 min after various interventions are shown in Fig 5. Compared to the sham control, enhanced activities were observed in MEP waveforms recorded 30 min after treatment with CES-iTBS, Opto-iTBS, and Opto-cTBS. Decreased activity in MEP waveform was found following CES-cTBS. Fig 6 shows fold changes of MEP activity at each time point following CES-TBS and Opto-TBS interventions. In Fig 6A, the effects of treatment and time-course were analyzed by Kruskal-Wallis test and Friedman’s test separately. First, to analyze the difference between CES-iTBS versus sham and CES-cTBS versus sham at each time-course, Kruskal-Wallis test with post hoc Dunn’s test were used. Comparing to sham, MEPs were significantly increased by CES-iTBS at 10 min and 30 min (#*p* < 0.05 versus sham). Then, the effect of time-course was analyzed by Friedman’s test for each type of treatment. Post hoc Dunn’s test revealed that the MEPs significantly increased at 15 min and remained at increased levels for up to 30 min (*p < 0.05 versus -10 min) after CES-iTBS compared to baseline MEP.

The TBS protocols were then converted into optogenetic stimulation to test the modulating effects of glutamatergic-specific stimulation on MEP activity. In Fig 6B, the effects of treatment were first analyzed by Kruskal-Wallis test plus post hoc Dunn’s test. Comparing to sham, Opto-iTBS increased MEPs from 5 min through 30 min after the treatment (#*p* < 0.05 versus sham). Then, the effect of time-course on MEPs was examined by Friedman’s test with post hoc Dunn’s test for each type of treatment. Results showed that when comparing to -10 min baseline, the MEPs significantly increased at 15 min and remained at increased levels for up to 30 min (*p < 0.05 versus -10 min) after both Opto-iTBS and Opto-cTBS. No significant after-effect was found on the MEP amplitudes in the sham group comparing to the baseline.

## Discussion and conclusion

We developed a modified optogenetic neural interface, which was integrated with CES and a cortical recording system, to achieve chronic *in vivo* cortical stimulation and monitoring in ChR2-expressed rats. It is highly desirable to simultaneously record electrophysiological responses during optogenetic activation for assessing brain neural networks. In the present study, the modified optogenetic neural interface employed an implanted fiber guide to target the brain region of interest after ChR2 expression (M1 in this case), and a stainless steel fiber cannula, which allowed CES and LFP recording via a dual-function electrode. LFP evoked by optical pulses was recorded using the cannula (contact area = 0.097 mm^2^) from the surface of the cortex simultaneously during optogenetic stimulation. The peak-to-peak amplitude of evoked potentials increased with increasing optical intensity (Fig 3B). Consistent amplitude and similar morphologies were found compared to those reported in a previous *in vitro* study [21].

Using this custom-made neural interface, TBS protocols used for inducing LTP- or LTD-like plasticity in human M1 were converted into an optogenetic animal model, and then optical and electrical control of the rodent’s motor activity was achieved. This study showed that single-pulse CES delivered through the custom-made optrode could elicit MEP. The obtained recordings were similar to those obtained using CES delivered through epidural screw electrodes [13]. By comparing the MEP amplitudes before and after TBS intervention, modulated neuroplasticity via cortical TBS can be observed. It was found that CES-iTBS induces potentiation whereas CES-cTBS inhibits MEP activity (Figs 5 and 6A). These observations are similar to those for TBS-induced LTP- or LTD-like plasticity reported in our previous studies using CES and rTMS [10,13].

The TBS protocols were converted into a focal optogenetic stimulation scheme to test the after-effects on motor plasticity. A ChR2 (H134R) variant and 1-ms stimulation pulses were used in the TBS protocols to ensure spiking fidelity. Since ChR2 (H134R) can respond to stimulation with frequencies of up to 80 Hz [22,23], Opto-TBS with a 50-Hz intra-burst frequency could reliably activate evoked potentials in M1 (Fig 4, upper right). We found that both Opto-iTBS and Opto-cTBS can increase MEP amplitudes. The enhancement of MEP activity by Opto-iTBS was stronger than that by CES-iTBS (Figs 5 and 6B). Fold change values of MEP measured at 30 min post-stimulation are: CES-iTBS: 2.57, CES-cTBS: 0.38, Opto-iTBS: 3.77, and Opto-cTBS: 2.21. The potentiating effect of the Opto-iTBS protocol on MEP activity is comparable to that for the CES- and rTMS-iTBS protocols. However, a discrepancy was observed in the case of cTBS treatment: CES- or rTMS-cTBS suppressed MEP activity whereas Opto-cTBS enhanced it. Unlike CES- or rTMS-cTBS, which typically suppressed the MEP response, Opto-cTBS enhanced it at a level similar to that of CES-iTBS.

It is hypothesized that the inconsistency between potentiation caused by Opto-cTBS versus suppression caused by CES/rTMS-cTBS may have arisen from the difference in neural networks targeted by these two TBS modalities. Some studies have indicated that the after-effects of iTBS and cTBS are mediated by different interneuron networks excited by rTMS [5,11]. Research has also shown that different TMS coil orientations can activate different intra-cortical networks, leading to approximately 50% of the variation in TBS response [24]. These results indicate that the response to TBS treatment is strongly influenced by the interneuron networks recruited by the TMS pulse. There is thus a need for a focalized brain stimulation technique to selectively target neural pathways with a specific cell population. In the present study, we tested the after-effects of cell-type-specific TBS using the optogenetic approach. Since ChR2 was expressed specifically in excitatory neurons (such as pyramidal neurons) driven by the CaMKIIα promoter, Opto-TBS should excite primarily the pyramidal neurons in a focalized area, in contrast to CES/rTMS-TBS, which activates all types of neuron, including interneurons. Therefore, it is hypothesized that the opposite modulation of neuroplasticity by Opto-cTBS compared to that of CES-cTBS may be attributed to the priming effects on different neuronal populations recruited by optogenetic and electrical/magnetic pulses. It is known that rTMS and CES approaches excite all types of cell and that optogenetic stimulation targets specific cells. Under rTMS or CES without targeting of specific cells, cTBS may suppress after-effect on cortical excitability through interneuron network. In contrast, Opto-cTBS may enhance cortical excitability when optogenetic stimulation targets excitatory neurons specifically. The major limitation of our study is that other opsin tools, such as GABAergic neurons, were not used to target interneurons in the cortex to investigate the modulating effects of TBS mediated by interneurons.

In conclusion, we developed a custom-made optogenetic neural interface, which can perform cortical recording to test the after-effects of a TBS scheme on motor plasticity in ChR2-expressing rats. Our study showed the feasibility of using the optogenetic approach for focalized and cell-type-specific modulation of neuroplasticity. Using the custom-made neural interface, TBS protocols used for testing LTP- or LTD-like plasticity in the human M1 were converted into an optogenetic animal model. It was confirmed that the potentiating effect of the Opto-iTBS protocol on MEP activity is comparable to that of CES- and rTMS-iTBS protocols. However, a discrepancy was observed in the case of cTBS treatment: CES- or rTMS-cTBS suppressed MEP activity whereas Opto-cTBS enhanced it. The results provide a better understanding of TBS-induced brain modulation and encourage further exploration of cell-type-specific brain stimulation.

## Abbreviations

CES: cortical electrical stimulation
ChR2: channelrhodopsin-2
cTBS: continuous theta burst stimulation
iTBS: intermittent theta burst stimulation
LFP: local field potential
LTD: long-term depression
LTP: long-term potentiation
M1: primary motor cortex
MEP: motor-evoked potential
NIBS: non-invasive brain stimulation
Opto-cTBS: optogenetic continuous theta burst stimulation
Opto-iTBS: optogenetic intermittent theta burst stimulation
rTMS: repetitive transcranial magnetic stimulation
TBS: theta burst stimulation
